# Hydrophobia

**Published:** 1889-10

**Authors:** J. J. Llack

**Affiliations:** Friar’s Point, Miss.


### HYDROPHOBIA.

I have just attended a case, which on account of its infrequent occurrence, may be of sufficient interest to the profession, to report.

Sunday, June 23, 11 A. M., I was called to see Julius Gill, a colored boy, aged twelve years. His mother said he had been complaining since Saturday noon, would eat nothing, had "choking spells" and could not swallow. Boy said his throat was sore, and head pained. On examination, I found considerable hyperemia of fauces, tonsils a little enlarged and œdematous; pulse 120, respiration 36, temperature 100°, and very nervous.

Prepared a simple sedative mixture, and on offering it, he was seized with sudden trembling, eyes seemed starting from their sockets, gulped at the fluid, followed by violent pharyngeal spasm, with complete inability to swallow. The symptoms were so peculiar and suggestive that my attention was at once called to hydrophobia, and upon inquiry if the boy had been bitten, drew out the following history, which enabled me at once to diagnose that disease.

Eight weeks previous, a dog, supposed to be mad was seen to bite the Gill dog and though shot at several times escaped. The family were told of the occurrence, but contented themselves with tying up their dog for a while. He broke the rope and left and was absent several days when he returned looking very much emaciated and sick.

The patient attempted to fondle him and was bitten severely on the left cheek. The dog was immediately killed. The wound was not cauterized but quickly healed and gave no trouble.

I gave fluid ext. gelsemium five drops, antifebrine five grains, in a teaspoon of water. Spilled about half, but patient was quieted. I then prepared:

| | | |
|---|---|---|
| ℞ | Ext. gelsem fl. | 24 drops. |
| | Potass. brom. | 30 grains. |
| | Chlor. anodyne | 30 drops. |
| | Aquæ pura q. s. ad. | 1 ounce. |

M. Sig. Alternate with:

| | | |
|---|---|---|
| ℞ | Antifebrine | 20 grains. |
| | Hydg. cum cretæ | 20 grains. |
| | Cinchonidia | 10 grains. |

M. et. ft. chart 4. Sig. One every two hours.

I saw the patient at 3:30 p. m., in company with Dr. J. A. Cooper, whom I invited to study the case with me on account of its infrequency. Found him quiet, pulse 120, temperature 99°, respiration 36. Reflex irritability somewhat diminished under the sedative. Solids could be swallowed with an effort, but liquids with the greatest difficulty, though thirsty. Bowels confined. Respiration costal, with a peculiar catch about every half minute. Neck stiffened, and constant effort was being made to clear the throat from ropy, tenacious saliva which accumulated in great quantities.

As Dr. C. had not seen the reflex spasms very well marked, and as he thought the free flow of saliva was caused by the gelsem., I withdrew that mixture, in order to free him from its influence, and put him on:

| | | |
|---|---|---|
| ℞ | Potass. brom. | 60 grains. |
| | Tinct. belladon. | 10 drops. |
| | Aquæ pura | 1 ounce. |

M. Sig. One teaspoonful.

At 6 p. m., was recalled. Reflex paroxysms very strongly developed. Could force, with the greatest difficulty, a few drops of water at a time. Fanning produced the same results as an effort to swallow. Temperature 100°, respiration 38, shallow; catch very prominent; pulse 120, and very feeble and irregular. Resumed the gelsemium which seemed to quiet. Bowels had not acted.

At 9 p. m., visited patient in company with Dr. C.; quiet. Temperature 101°, respiration 38, frequent sighing, pulse 120, irregular. Flow of saliva lessened. Continued the antifebrine and bromide.

9:30 a. m., June 24. Temperature 101°, pulse 120, respiration 35, sighing more frequent. Patient very restless and garrulous, eyes bright, mind wandering. Reflex paroxysms very strong on attempt to drink, slept none previous night. Urine diminished in quantity and highly colored. 3 p. m. Symptoms unchanged. Respiration 36, temperature 101½°, pulse 120, very weak. Opened bowels with enema glycerin, one grain, more quiet afterward. 9 p. m. Pulse 120, very weak, first sound of heart distinct, but second scarcely observable.

Respiration 38, costal, sighing frequent, temperature 103¾°. Reflex irritability of skin was increased so much that fanning brought on terrible paroxysms and patient was wild with fright but soon became quiet when fanning was discontinued.

Paroxysms of shouting and singing in the monotonous tone common to negroes. Mother had been telling him to pray and encouraged him in shouting.

Flow of urine free but diminished in quantity. Ate a bowl of bread softened in milk, swallowing solid part with a gulp, but avoiding fluid part. On attempting to drink would spring up from bed and a violent paroxysm would follow. The sound of water poured from one vessel to another, and mirror shown him did not seem to effect him perceptibly.

6 a. m., June 25. Patient had got up and dressed himself. Too restless to take temperature. Pulse absent at wrist. Respiration quick and shallow with the "catch" coming more frequently. Heart very weak. Mind wandering, but knew those around him. Skin having a water-soaked appearance, and wet with a cool, sticky perspiration. Would not attempt to drink. Paroxysms of shouting continued, though very weak.

Death occurred at 7:30 from exhaustion.

If any of the profession wish to ask any questions I shall be happy through the Brief or by letter.—J. J. LLACK, M. D., of Friar's Point, Miss., in *Medical Brief.*

